# Supplementary material for: Intention to learn modulates the impact of reward and punishment on sequence learning
Source: Sci Rep. 2020 Jun 1;10:8906. doi: 10.1038/s41598-020-65853-w (PMC7264311; doi:10.1038/s41598-020-65853-w)
Supplement: Supplementary file 1 — Supplemental table 1. [file 41598_2020_65853_MOESM1_ESM.docx]

**Supplemental information: Intention to learn modulates the impact of reward and punishment on sequence learning**

Adam Steel^1,2^, Chris I. Baker^2,$^, Charlotte J. Stagg^1,3$^

^1^Wellcome Trust Centre for Integrative Neuroimaging, FMRIB, Nuffield Department of Clinical Neurosciences, University of Oxford, Oxford, UK

^2^Laboratory of Brain and Cognition, National Institute of Mental Health, National Institutes of Health, Bethesda, MD, 20814

^3^ Oxford Centre for Human Brain Activity (OHBA), Wellcome Trust Centre for Integrative Neuroimaging, University Department of Psychiatry, University of Oxford, Oxford, UK

^$^ Joint Senior Authors

Corresponding Author: Adam Steel, Wellcome Trust Centre for Integrative Neuroimaging, FMRIB, John Radcliffe Hospital, Headington, Oxford, OX3 9DU, United Kingdom; Tel. +1 (202) 640 9340; current email: [adam.steel@dartmouth.edu](mailto:adam.steel@dartmouth.edu)

## Supplemental information

Table 1. Accuracy data for the intentional and unintentional experiments.

| **Accuracy** |  |  |  |  |
| --- | --- | --- | --- | --- |
| **Unintentional** |  |  |  |  |
| **Block** | **Sequence-type** | **Condition** | **Accuracy (%)** | **Stdev** |
| 0 | Fixed | Reward | 0.964 | 0.024 |
|  |  | Punishment | 0.96 | 0.037 |
|  |  | Control | 0.972 | 0.027 |
|  | Random | Reward | 0.948 | 0.033 |
|  |  | Punishment | 0.937 | 0.049 |
|  |  | Control | 0.944 | 0.04 |
| 1 | Fixed | Reward | 0.944 | 0.065 |
|  |  | Punishment | 0.948 | 0.036 |
|  |  | Control | 0.976 | 0.023 |
|  | Random | Reward | 0.944 | 0.031 |
|  |  | Punishment | 0.929 | 0.053 |
|  |  | Control | 0.936 | 0.039 |
| 2 | Fixed | Reward | 0.953 | 0.041 |
|  |  | Punishment | 0.932 | 0.039 |
|  |  | Control | 0.964 | 0.042 |
|  | Random | Reward | 0.931 | 0.044 |
|  |  | Punishment | 0.927 | 0.059 |
|  |  | Control | 0.938 | 0.049 |
| 3 | Fixed | Reward | 0.955 | 0.032 |
|  |  | Punishment | 0.922 | 0.053 |
|  |  | Control | 0.957 | 0.043 |
|  | Random | Reward | 0.931 | 0.05 |
|  |  | Punishment | 0.908 | 0.043 |
|  |  | Control | 0.948 | 0.026 |
| 4 | Fixed | Reward | 0.938 | 0.025 |
|  |  | Punishment | 0.922 | 0.056 |
|  |  | Control | 0.953 | 0.039 |
|  | Random | Reward | 0.913 | 0.061 |
|  |  | Punishment | 0.891 | 0.072 |
|  |  | Control | 0.946 | 0.034 |
| 5 | Fixed | Reward | 0.929 | 0.061 |
|  |  | Punishment | 0.918 | 0.061 |
|  |  | Control | 0.957 | 0.037 |
|  | Random | Reward | 0.934 | 0.043 |
|  |  | Punishment | 0.906 | 0.047 |
|  |  | Control | 0.953 | 0.027 |
| 6 | Fixed | Reward | 0.939 | 0.071 |
|  |  | Punishment | 0.931 | 0.05 |
|  |  | Control | 0.944 | 0.04 |
|  | Random | Reward | 0.915 | 0.066 |
|  |  | Punishment | 0.877 | 0.077 |
|  |  | Control | 0.939 | 0.047 |
| 7 | Fixed | Reward | 0.95 | 0.037 |
|  |  | Punishment | 0.946 | 0.039 |
|  |  | Control | 0.948 | 0.046 |
|  | Random | Reward | 0.915 | 0.05 |
|  |  | Punishment | 0.889 | 0.07 |
|  |  | Control | 0.931 | 0.054 |
| **Intentional** |  |  |  |  |
| **Block** | **Sequence-type** | **Condition** | **Accuracy (%)** | **Stdev** |
| 0 | Fixed | Reward | 0.944 | 0.051 |
|  |  | Punishment | 0.977 | 0.033 |
|  |  | Control | 0.944 | 0.05 |
|  | Random | Reward | 0.929 | 0.037 |
|  |  | Punishment | 0.944 | 0.038 |
|  |  | Control | 0.95 | 0.036 |
| 1 | Fixed | Reward | 0.941 | 0.042 |
|  |  | Punishment | 0.946 | 0.031 |
|  |  | Control | 0.953 | 0.043 |
|  | Random | Reward | 0.917 | 0.063 |
|  |  | Punishment | 0.901 | 0.069 |
|  |  | Control | 0.931 | 0.046 |
| 2 | Fixed | Reward | 0.931 | 0.029 |
|  |  | Punishment | 0.943 | 0.03 |
|  |  | Control | 0.957 | 0.03 |
|  | Random | Reward | 0.932 | 0.031 |
|  |  | Punishment | 0.931 | 0.051 |
|  |  | Control | 0.943 | 0.045 |
| 3 | Fixed | Reward | 0.936 | 0.054 |
|  |  | Punishment | 0.944 | 0.032 |
|  |  | Control | 0.951 | 0.034 |
|  | Random | Reward | 0.905 | 0.058 |
|  |  | Punishment | 0.906 | 0.064 |
|  |  | Control | 0.927 | 0.044 |
| 4 | Fixed | Reward | 0.911 | 0.042 |
|  |  | Punishment | 0.937 | 0.047 |
|  |  | Control | 0.955 | 0.025 |
|  | Random | Reward | 0.918 | 0.055 |
|  |  | Punishment | 0.894 | 0.054 |
|  |  | Control | 0.927 | 0.042 |
| 5 | Fixed | Reward | 0.924 | 0.054 |
|  |  | Punishment | 0.948 | 0.059 |
|  |  | Control | 0.965 | 0.03 |
|  | Random | Reward | 0.891 | 0.053 |
|  |  | Punishment | 0.892 | 0.056 |
|  |  | Control | 0.946 | 0.044 |
| 6 | Fixed | Reward | 0.931 | 0.036 |
|  |  | Punishment | 0.934 | 0.038 |
|  |  | Control | 0.955 | 0.031 |
|  | Random | Reward | 0.885 | 0.046 |
|  |  | Punishment | 0.91 | 0.058 |
|  |  | Control | 0.925 | 0.042 |
| 7 | Fixed | Reward | 0.929 | 0.047 |
|  |  | Punishment | 0.927 | 0.061 |
|  |  | Control | 0.96 | 0.04 |
|  | Random | Reward | 0.894 | 0.055 |
|  |  | Punishment | 0.898 | 0.044 |
|  |  | Control | 0.939 | 0.035 |
